# Biofortification of Mushrooms: A Promising Approach

**DOI:** 10.3390/molecules29194740

**Published:** 2024-10-07

**Authors:** Klaudia Słyszyk, Marek Siwulski, Adrian Wiater, Michał Tomczyk, Adam Waśko

**Affiliations:** 1 Department of Biotechnology, Microbiology and Human Nutrition, Faculty of Food Science and Biotechnology, University of Life Sciences in Lublin, ul. Skromna 8, 20-704 Lublin, Poland; klaudia.kowalik@up.lublin.pl; 2 Department of Vegetable Crops, Poznań University of Life Sciences, ul. Dąbrowskiego 159, 60-594 Poznań, Poland; marek.siwulski@up.poznan.pl; 3 Department of Industrial and Environmental Microbiology, Institute of Biological Sciences, Faculty of Biology and Biotechnology, Maria Curie-Skłodowska University, ul. Akademicka 19, 20-033 Lublin, Poland; adrian.wiater@mail.umcs.pl; 4 Department of Pharmacognosy, Faculty of Pharmacy with the Division of Laboratory Medicine, Medical University of Białystok, ul. Mickiewicza 2a, 15-230 Białystok, Poland; michal.tomczyk@umb.edu.pl

**Keywords:** biofortification, mushroom, hidden hunger, nutrition, humans

## Abstract

Mushrooms exhibit a broad spectrum of pharmacological activities and are widely used for medical purposes and in nutrition. Numerous bioactive metabolites are responsible for these activities. Their distribution and biological effects differ depending on the fungal species and their chemical composition. Biofortification is a sustainable process that aims to improve the nutritional profile of food crops, as most of them are low in key nutrients. This review aims to delve into the process of fungal biofortification and review the most commonly used elements and species. Through biofortification, it is possible to combat hidden hunger, which affects as many as 2 billion people worldwide. “Hidden hunger” is a phenomenon in which the organism lacks the minerals and vitamins needed for development, growth, and good overall health. Mushrooms are increasingly being considered for biofortification due to their ability to accumulate various elements (both micro- and macroelements).

## 1. Introduction

Biofortification is a process of enriching mushrooms or plants with certain elements, by which the nutritional quality of food crops is improved through agronomic practices, conventional plant breeding, or modern biotechnology. It involves the accumulation or synthesis of nutrients at the source. The bioavailability of nutrients is a phenomenon linked to biofortification. Compared to standard fortification, biofortification can increase bioavailability. Biofortification can increase the bioavailability of nutrients by increasing the concentration of nutrients, changing chemical forms to ones that are more easily absorbed by the body, increasing the content of absorption enhancers such as polyphenols, and interacting with other nutrients that can synergistically promote the absorption of others [1]. The consumption of such enriched food is aimed at reducing the incidence of diseases caused by elemental deficiencies. This process provides an excellent way of producing the so-called functional food. It can contribute to the prevention of diseases and has a beneficial effect on health. The biofortification method has been recognised in recent years as one of the fastest, cheapest, and most natural methods that can improve the mineral richness of foods [2]. As far as plants are concerned, wheat, rice, fruit, potatoes, carrots, tomatoes, and lettuce can be improved with this method. In turn, a popular fungal species used in biofortification are mushrooms of the genus *Pleurotus* (Fr.) P. Kumm. In determining the potential for biofortification, the effects of the chemical form of the elements are studied. Their dosage and application routes, as well as the effects of single elements and their mixtures, are assessed. Biofortification should not lead to a reduction in plant yield or quality [3]. Humans need at least 22 elements for proper development. Worldwide, around 2 billion people suffer from a lack of essential nutrients. This problem is related to the insufficient uptake of nutrients from soils and their consequent incorporation into the food chain [4]. One of the causes of the low mineral content in plants is the deficiency of minerals in soils where crops are grown. Another cause may be that nutrients in soils may be present in forms that are difficult to assimilate and inaccessible to plants or fungi. The World Health Organisation has programmes aimed at combating mineral deficiency, which is a direct cause of bodily dysfunction. The deficiency may also be the cause of a phenomenon known as ‘hidden hunger’. This problem affects approximately 28% of people worldwide [5]. Mushrooms are increasingly being considered for biofortification due to their ability to accumulate various elements. Therefore, they can be a good option for enriching foods with the micro- and macroelements necessary for the proper functioning of the human body [6]. During their growth period, mushrooms have the ability to absorb and bioaccumulate mineral elements in the form of organic molecules. Therefore, mushrooms can be used to supply nutrients that are not provided in the human diet in sufficient proportions. Some of these vital elements that mushrooms may bioaccumulate include iron, zinc, copper, selenium, etc. [7]. In general, the biofortification of mushrooms offers many benefits, such as a higher content of a particular element and improving the nutritional and biochemical profiles of the fungi (enhancement of antioxidant properties or improvement in the total protein content) [8]. Selenium-biofortified mushrooms have additional anticancer and antioxidant properties. The consumption of such mushrooms also brings benefits in the treatment and prevention of cardiovascular, neurodegenerative, and immunological diseases, HIV infection, cancer, or senescence [9]. Another source of highly available lithium with enormous potential is the enhanced *Pleurotus ostreatus* fungus. In comparison to psychiatric medications containing Li_2_CO_3_, the high mineral content in fungal biomass was linked to a better availability being achieved after sequential extraction and in vitro digestion [10].

**Figure 1:**
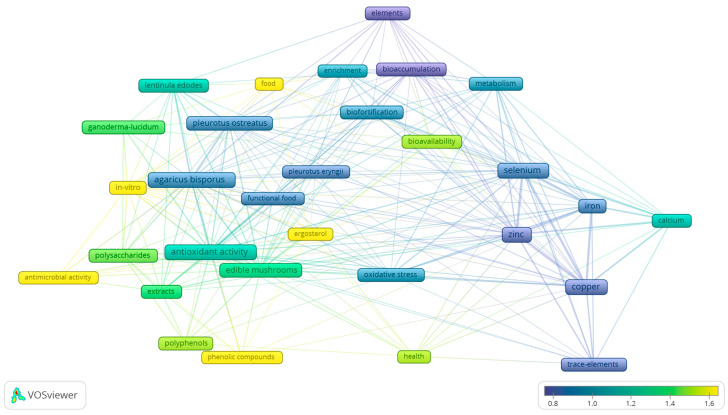
Visualisation of VOSviewer results on the biofortification of edible mushrooms published from December 2018 to January 2024, with key terms ‘biofortification’, ‘edible mushrooms’, ‘elements’, ‘antioxidant activity’, ‘functional food’, ‘nutrition’, and ‘nutritional compounds’.

The analysis of researchers’ interest in the topic of fungi revealed that antioxidant properties, antimicrobial properties, and bioavailability of compounds were studied most comprehensively (Figure 1). Phenolic compounds, polysaccharides, and ergosterol turned out to be the most frequently investigated compounds. The biofortification of fungi with various micronutrients and macronutrients was of interest to researchers but to a somewhat lesser extent. Nevertheless, there was an evident upward trend and increasing interest in this topic over the years. *Pleurotus ostreatus* (Jacq.) P. Kumm., *P. eryngii* (DC.) Quél, *Agaricus bisporus* (J.E. Lange) Imbach, *Lentinula edodes* (Berk.) Pegler, and *Ganoderma lucidum* (Curtis) P. Karst. are the most commonly analysed fungal species. The review explores the process of fungal biofortification, highlighting commonly used species and discussing recent advancements in technology for this purpose.

## 2. Search Methodology

To conduct this systematic review, the specialised electronic databases PubMed, SCOPUS, EMBASE, or REAXYS were searched for manuscripts on edible mushroom biofortification published over the previous 6 years from December 2018 to January 2024. The terms “biofortification”, “edible mushrooms”, “elements”, “antioxidant activity”, “functional foods”, “nutrition”, and “nutritional compounds” were combined to perform the search. Based on a study of titles and abstracts, a review of the retrieved papers was carried out. Only published English-language and Polish-language research articles were chosen.

## 3. Nutritional Composition of Mushrooms

Due to their unique flavour, mushrooms have very often been considered part of the human diet. They are also used in folk medicine. In recent decades, many bioactive substances have been isolated from mushrooms, hence the increased interest in fungal research. Currently, mushrooms are being used to produce functional foods. To date, 140,000 species of fungi have been identified worldwide. However, it is estimated that their total number is between 2.2 and 3.8 million. [11]. Of the species described, approximately 21,000 are *Macromycetes*. Approximately 2000 species of macrofungi are edible mushrooms. Currently, around 200 species of mushrooms collected from natural sites are used for culinary and medicinal purposes [12]. The nutritional value of mushrooms consists of the presence of proteins, fatty acids, saccharides, dietary fibre, vitamins, and minerals in their composition. Mushrooms are characterised by their high protein content. Mushroom protein has a good bioavailability level of up to 90%. Additionally, fruiting bodies contain 4.7–6.9% of total saccharides, of which 2.7–3.9% is dietary fibre. The fatty acid content is low, but the ratio of polyunsaturated to saturated fatty acids is considered favourable [13]. Mushrooms contain many minerals, such as phosphorus, potassium, magnesium, and calcium. They are also a good source of trace elements, mainly zinc, copper, iron, selenium, molybdenum, and magnesium. The main biologically active substances found in mushrooms include polysaccharides, sesquiterpenes, glycoproteins, and triterpenoids, which determine their immunomodulating and anticancer effects. Mushrooms also contain natural antibiotics, statins, and antioxidants, including phenolic compounds, flavonoids, carotenoids, and tocopherols [14]. Despite their slightly lower nutrient content, many wild mushroom species contain higher levels of antioxidants than cultivated species [15,16]. Species of mushrooms with the highest content of antioxidant compounds include *Boletus edulis* and *Cantharellus cibarius*. Mushrooms have many health-promoting properties, e.g., anticancer activity (*Boletus edulis*, *Lactarius deliciosus*), antioxidant activity (*Armillaria mellea*, *Leccinum aurantiacum*, *Suillus luteus*, *Xerocomus badius*), anti-inflammatory activity (*B. edulis*, *Morchella esculenta*), antimicrobial activity (*B. edulis*, *C. cibarius*, *L. deliciosus*), antiviral activity (*B. edulis*), hypoglycaemic activity (*B. edulis*, *C. cibarius*), and neuroprotective activity (*C. cibarius*, *Tricholoma equestre*) [17]. Edible mushrooms are renowned for their unique taste. Unfortunately, many people still believe that they have no nutritional value. In fact, edible mushrooms are a source of substances that are valuable to health and have an impact on the body’s normal function. Table 1 compares popular edible mushrooms with other foods that are equally often consumed. As can be seen, mushrooms contain a number of valuable minerals and can be consumed similarly to other foods.

## 4. Biofortification Techniques

**Figure 2:**
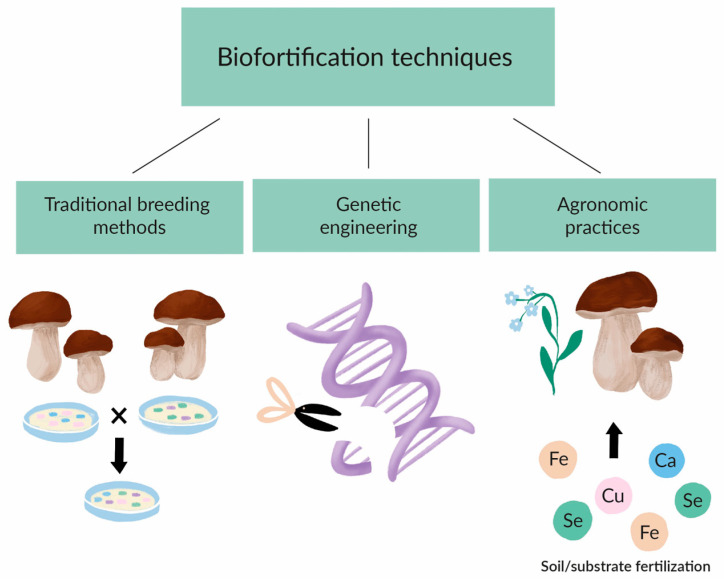
Types of biofortification.

Biofortification is one of the numerous methods and strategies that have been recommended to combat micronutrient deficiencies, which can be achieved by three different methods: traditional breeding methods, genetic engineering, and agronomic practices (Figure 2).

Significantly, biofortification is often regarded as a sustainable, economical, and successful way to increase the amount of micronutrients in diets. Unlike fortification, which adds nutritional additives to food during food processing, biofortification aims to naturally increase the nutritional value of foods. In this way, health improvement can take place by preventing disease through nutrition rather than drugs. The major constraints, including the stability, storage, legal concerns, and biosafety of biofortified crops, still need to be taken into account despite extensive studies [18].

By carefully combining many varieties to select certain qualities, traditional breeding methods can increase the nutritional value of crops. To generate a hybrid with better qualities, the method entails choosing organisms that have desired properties, such as a higher micronutrient content, and crossing them with others. This procedure is done over time, and the progeny are examined for the desired characteristics. When crops naturally contain certain amounts of micronutrients or when the genetic variety is available in a form that can be used, biofortification through breeding is achieved. Some of the most effectively biofortified cultivars have been produced through conventional breeding by large-scale research initiatives across the world, including the BioCassava Plus programme, the HarvestPlus global programme, and the Health Grain Project of the European Union [19]. Biofortified crops include beans high in iron, rice high in zinc, rice high in selenium, and sweet potatoes high in iodine and vitamin A. Since it is safe and does not raise the same safety issues as genetic engineering, this approach is widely accepted. Nevertheless, producing a crop with an elevated nutritional content may take several years due to the labour-intensive and slow nature of conventional breeding [8].

Biotechnology is a scientific area that involves the use of live organisms, cells, or their constituent parts to yield a valuable product. Many of the most urgent issues facing the planet might be resolved based on biotechnological solutions, e.g., the production of enough food to support the world’s expanding population, the development of novel disease cures, and the improvement of the nutritional value of crops. Compared to conventional methods, genetic engineering facilitates a more accurate and effective targeting of particular nutrients. The nutrient content can be increased by increasing the expression of already present genes that are involved in nutrient production or by introducing additional genes responsible for higher levels of vitamins or minerals. Genes that enhance the concentration and bioavailability of micronutrients and those that block antinutritional factors in crops limiting nutrient uptake can all be included concurrently using transgenic procedures [20]. A sensible way to increase the concentration and bioavailability of micronutrients is the use of transgenic technology, particularly in cases where crops have little genetic diversity. One such example is the engineering of “golden rice”, i.e., vitamin A-rich rice that may aid in the treatment of vitamin A insufficiency in underdeveloped nations. This method is controversial and is not recognised around the world due to the traditional way of thinking [2].

Agronomic biofortification is a process of enriching plants or fungi through the soil, growing substrate management, and fertilisation. It is the cheapest and fastest efficient way to produce food rich in macronutrients and micronutrients. Unfortunately, it provides a short-term solution [21]. It is based on the use of inorganic and organic fertilisers or biofertilisers. The source of the fertiliser used, the dose of the fertiliser, the transport of nutrients, and the application stage all have an impact on the final concentration of micronutrients. The success of agronomic biofortification is highly variable due to differences in the mobility and accumulation of minerals and the soil composition in a specific geographical area of cultivation [3]. Chelate fertilisers, water-soluble fertilisers, and nano-fertilisers with high uptake efficiency and a greater translocation of minerals have proved to be innovative approaches that have increased the success of biofortification. Interactions between nutrients may affect the effectiveness of this method. Some micro- and macroelements can work synergistically, increasing the absorption and utilisation of other elements. Another example may be nutrients that compete with each other for absorption. An innovative technique could be the simultaneous strengthening of crops with more than one element [22].

## 5. Species of Biofortified Mushrooms

The analysis of the literature data showed that the genus *Pleurotus* is the most commonly considered fungi for biofortification, and selenium is the most often added element. The species and elements used in the biofortification process are shown in Table 2.

The *Pleurotus* species are easy to cultivate due to their rapid growth and 100% biological productivity. The presence of high mineral content in the *Pleurotus* species is considered an alternative source of elements besides meat, fish, and vegetables [56]. Cordycepin, a major active ingredient in *Cordyceps militaris*, has a variety of biological activities and a number of pharmacological characteristics that are crucial for cancer immunotherapy [23,57]. *Lentinus crinitus* exhibits strong antioxidant activity and has the ability to bioaccumulate lithium in the mycelial biomass, which confers its great health-promoting benefits [58]. The main bioactive component of *Coriolus versicoloris* is a polysaccharopeptide (PSP). PSP increases the expression of cytokines and chemokines, such as tumour necrosis factor α (TNF-α), interleukins (IL-1β and IL-6), prostaglandin E, and histamine. It also enhances macrophage phagocytosis. The *Grifola frondosa* fungus generates a wide range of bioactive compounds. β-glucans have been found to be the main bioactive components responsible for anticancer activity [59]. The polysaccharide derived from *Phiolota nameko* has been found to have anti-inflammatory, immunological, antihyperlipidaemic, and antioxidant properties. Nevertheless, the use of its active components is severely limited by its poor water solubility [60]. *Ganoderma lucidum* holds considerable promise to help heal diseases that now plague the world’s population, such as diabetes, neurodegenerative illnesses, atherosclerosis, and inflammation. A broad range of pharmacological antioxidant, immunomodulatory, anti-neurodegenerative, antidiabetic, anti-inflammatory, anticancer, and antibacterial activities are demonstrated by polysaccharides derived from this mushroom [61,62]. *Schizophyllum commune* is a wood-degrading fungus. It is a primary source of schizophyllan (SPG), a β-glucan with a wide range of health-promoting qualities and industrial uses, particularly in the food, pharmaceutical, and cosmetics industries [63]. Lentinan (LNT) is the main bioactive component in *Lentinus edodes*. The anticancer properties of LNT are reflected in its ability to enhance the body’s immune system through various signalling pathways, thus exerting anticancer effects, even if it does not directly destroy cancer cells in the body [64]. *Agaricus bisporus* and *A. subrufescens* are the most widely consumed edible mushroom species worldwide. The composition of *A. bisporus* includes ergothioneine and antibiotic-related compounds such as benzoquinone derivatives. The tyrosinase isolated from this species shows a striking similarity to human tyrosinase. This strongly suggests that this species may have an abundant tyrosinase amount for use in medicine and cosmetics [65,66]. *A. subrufescens* has shown immunomodulatory and antimutagenic properties, although it is still unclear what chemical compounds and cellular mechanisms underlie its pharmacological effects [67]. Polysaccharides from *Flammulina velutipes* (FVP) are the main active ingredients, with a wide range of biological effects, including boosting immunity, enhancing cognition, having antioxidant properties, fending against ageing, and reducing tumour growth [68]. *Hericium erinaceus* produces at least 70 different bioactive metabolites, including β-glucans, erinacines, hericenones, sterols, alkaloids, and volatile aromatic compounds. Given its biological constituents, *H. erinaceus* has several health-promoting properties, including antimicrobial, antioxidant, anticancer, anti-fatigue, anti-ageing, neuroprotective, antidepressant, and anti-anxiety effects [69]. L-ergothioneine is a water-soluble thiol with cytoprotective and antioxidant properties. In particular, hypsin from *Hypsizygus marmoreus* is a thermostable ribosome-inactivating protein that can be obtained from the fungal fruiting body and has been shown to have antifungal and antiproliferative properties. Marmorin is another ribosome-inactivating protein that also inhibits HIV-1 reverse transcriptase and has antiproliferative effects [70]. *Inonotus obliquus* inhibits HIV-1 protease activity, regulates the immune system, eliminates free radicals, inhibits lipid peroxidation, suppresses symptoms of acute colitis, and regulates the serum levels of Th1/Th2 hormones [71]. The physiologically active constituents of *I. obliquus* include triterpenoids, ergosterol, ergosterol peroxide, sesquiterpenes, benzoic acid derivatives, hispidin, polysaccharides, and melanins [72]. *Poria cocos* fungi are mostly composed of triterpenes, polysaccharides, steroids, amino acids, choline, and histidine. In recent decades, significant progress has been achieved in the chemical and bioactive analyses of *P. cocos* polysaccharides (PCP) and their derivatives [73]. *Laetiporus sulphureus* secondary metabolites have anti-thrombin, antioxidant, and antibacterial properties. Non-carotenoid polyenelaetiporic acids found in *L. sulphureus* can be used as food-colouring agents in place of some manufactured ones [74,75]. FIP-vvo is the name given to an immunomodulatory protein (FIP) that was isolated from *Volvariella volvacea*. While numerous FIPs have been shown to regulate immunity, it is unknown how FIP-vvo affects the dendritic cells (DCs), which are essential in bridging the gap between innate and adaptive immunity [76]. A water-soluble polysaccharide with cytotoxic and immunostimulatory qualities was produced by *Calocybe indica*. By restoring antioxidant enzyme activity and lowering lipid peroxidation, the *C. indica* polysaccharide demonstrated significant antioxidant benefits and an anti-ageing impact in d-galactose-induced ageing rats [77]. The representatives of the genus *Agrocybe* contain a wide range of biological macromolecules, including lectins and polysaccharides, as well as a diverse group of small molecular chemical constituents categorised into sterols, sesquiterpenes, volatile compounds, polyenes, and other compounds [78,79].

The global market for mushroom production has grown significantly, with the industry valued at over USD 96.58 billion in 2023. Mushrooms are widely appreciated for their nutritional value and are consumed around the world, with the most cultivated and commercialised species being *Agaricus bisporus*, *Pleurotus* spp., and *Lentinula edodes.* Additionally, mushrooms are considered a delicacy due to their nutritional, medicinal, and organoleptic characteristics, and they provide numerous vitamins and minerals, making them a desirable food source [80]. Mushroom farming has the potential to help in the transition towards a circular agricultural economy by recycling agricultural wastes and turning them into high-quality products [81]. Thus, biofortified mushrooms may have great potential to help humanity in reducing deficiencies of certain elements.

## 6. Bioavailability of Nutrients in Biofortified Mushrooms

The route of mineral enrichment varies between different mushrooms. In edible mushrooms, mineral absorption mostly occurs through the mycelium. Different electrochemical potentials cause the passive movement of minerals across the plasma membrane. Furthermore, depending on the carriers on the protoplasmic membrane, minerals can potentially enter the fungal cell actively by requiring energy to enter through the membrane. Mushrooms are enriched with minerals mainly through active modes of transport, which is why edible mushrooms are able to accumulate a higher mineral content than plants [82].

### 6.1. Selenium and Zinc

Edible mushrooms, compared to most animal and plant sources, are considered an ideal choice for selenium bioaccumulation due to their rapid growth, better Se bioconversion capacity, and lower cultivation cost. *Lentinus edodes*, *Hericium erinaceus*, *Agaricus bisporus*, and *Pleurotus florida* are suitable fungal species for biofortification with this element [23]. They can convert inorganic selenium into selenoamino acids. In order to evaluate Se-enriched mushrooms as a potential dietary supply of this element, it is necessary to determine the form and bioavailability of Se that is accumulated from growth substrates [26]. The chemical forms of Se have an impact on its absorption and bioavailability. There are several oxidation levels of selenium found in the environment: elemental selenium, selenite, selenide, and selenate [50]. Increases in the level of this element in the blood are caused by the greater absorption of organic forms of selenium (SeMet) in humans and animals compared to inorganic forms. Compared to selenite and selenium, these organic selenium compounds are more beneficial due to their lower toxicity [51]. A study conducted by Bhatia et al. examined the bioavailability of Se in vitro in *Pleurotus florida*. Their size-exclusion analysis (HPLC-ICP-MS) performed after in vitro simulated gastrointestinal digestion showed that all high-molecular-weight selenium-containing compounds were broken down to low-molecular-weight selenocompounds. The majority of the bioaccessible Se was SeMet (selenomethionine), which made up at least 65% of the bioaccessible Se and 73% of the total number of forms found. Selenium-biofortified *P. florida* seems to be an excellent bioavailable source of selenium [45]. Selenium-enriched yeast has been recognised as an important nutritional source of selenium, providing chemo-preventative benefits beyond those observed for selenomethionine supplementation. Studies suggest that supplementation with selenium-enriched yeast may offer chemo-preventative benefits, indicating its potential role in preventing chronic diseases such as cardiovascular disease, cancer, and neurodegenerative diseases [83]. The other interesting form of selenium that has been used to biofortify yeast is urea-selenium. Urea-selenium biofortification of yeast can lead to the accumulation of organic forms of selenium, such as selenomethionine, in the yeast biomass, making it a valuable source of protein and selenium biocomplexes. Also, urea-selenium has been shown to enhance bioavailability and reduce selenium emissions in the environment, potentially improving the nutritional quality of yeast. However, it should be remembered that selenium can be toxic at high concentrations, and excessive consumption of selenium can lead to adverse health effects, emphasising the importance of carefully managing selenium biofortification processes. In conclusion, biofortified yeast enriched with selenium has potential applications in producing dietary supplements for both animals and humans, indicating its relevance to the food and nutrition industries [84].

Zinc biofortification is often combined with selenium. The bioavailability of zinc, like selenium, is limited by many internal and external factors. Its transport into tissues, toxicity, and availability depends on the form taken [44]. As in the case of selenium, organic zinc has higher bioavailability and lower toxicity than inorganic forms. The mechanisms of accumulation of zinc (II) ions are very diverse. Studies indicate that the addition of Zn may be a promising approach, as supplementation with low concentrations of Zn increases mycelial growth rates and biomass production [46].

A relatively new approach to enriching fungi with selenium and zinc ions is the use of nanoparticles (SZMs) [85]. The methods for enriching mushrooms with zinc and selenium nanoparticles involve growing mycelium and fruit in substrates containing various concentrations of selenium and zinc, leading to increased levels of these elements in the mushrooms. Consuming mushrooms enriched with zinc and selenium nanoparticles may offer health benefits such as improved antioxidant capacity and potential antitumor activities. However, there are potential environmental implications and challenges associated with the process, including the need to carefully manage selenium concentrations to avoid adverse effects on mushroom growth and the environment [86].

### 6.2. Iron

Iron is considered the most difficult element to be added to fortified foods due to the different properties of its chemical forms. Due to differences in iron accumulation efficiency, the choice of products to be enriched must be well thought out. It is also important to consider bioavailability, as the success of enrichment depends on the absorption of the element. The fungus species, the chemical type of Fe added, and the metal content in the medium all have a major impact on the bioaccumulation effectiveness of Fe [24]. As a result, it is impossible to come to a unanimous decision about the optimum species, concentration, or type of iron supplementation. Every case needs to be handled differently. To achieve the highest efficiency, ideal circumstances must be identified before selecting the species to be enriched. With the current understanding of the potential, implementation of this type of biofortification seems quite possible [6]. The following forms were used in most studies: FeSO_4_·7H_2_O, FeCl_3_, and chelated iron in various concentrations. While iron is necessary for fungal metabolism, excessive amounts can lead to the generation of free radicals that impair fungal metabolism and reproduction through enzymatic inhibition, ion displacement or substitution, and cell membrane rupture. Moreover, the production of iron-protective chemicals may cause the metabolism to reorganise, lowering the energy required for fungal growth. This would account for the decreased growth of mycelial biomass in culture media when high concentrations of iron ions are added [25]. Typically, iron exists as an insoluble salt. The fungus has evolved a number of methods for iron uptake, including acidification of the substrate, conversion of ferric iron ions to the ferrous form, and secretion of hydroxamates, which are iron-chelating molecules. Fungi produce siderophores, which are high-affinity iron-chelating chemicals that help transport iron across cell membranes [34]. These include rhododotorulic acid, fusarins, coprogens, and ferrichromes. They are also capable of catalysing forms that are reactive with oxygen and damaging to cells. Manoproteins help the fungus absorb iron more efficiently and help siderophore iron chelates stay in the cell wall [40].

### 6.3. Calcium

Calcium accumulation in edible mushrooms is an effective way to increase the action of this element, as its organic form can be formed from the original inorganic calcium. The main sources of calcium in edible mushrooms are CaCl_2_, CaCO_3_, or Ca(NO_3_)_2_. While the formation of Ca has been extensively investigated, the mechanisms behind Ca enrichment, which are closely linked to absorption, transport, and metabolism, have only been the subject of a small number of studies. In terms of calcium enrichment in edible mushrooms, an increase in the calcium ion content causes an increase in the activity of the calcium transport system Ca^2+^-ATPase on the mycelial cell membrane. Progressive improvement in the rate of calcium transport and absorption has been reported [32]. Nevertheless, Ca^2+^-ATPase activity was hindered when the ion addition was raised to a threshold value. This reduced calcium absorption and transport led to an inadequate calcium buildup in the mycelium. Calcium may be passively transported through a non-specific channel in the cell membrane, diffused further through the mycelium, or transported extracellularly from the medium to the fruiting bodies via the interfungal cavity. In edible fungi, calcium ions attach themselves to reactive groups in such biomacromolecules as polysaccharides, proteins, nucleic acids, etc., finishing the process of converting inorganic calcium into its organic form [54]. In plants, the Ca uptake depends on the form and level of Ca and on the activity of a high- or low-affinity membrane transporter. It is still unknown, nevertheless, whether the Ca transporters found in edible fungi are the same as those found in plants and whether they are general or specialised transporters [87].

### 6.4. Lithium

Lithium is commonly used in psychiatric drugs because it blocks intracellular neurotransmission enzymes in the central nervous system [49]. While several metals are necessary for the growth and metabolism of fungi, greater amounts of these elements can be harmful. Adsorption in the cell wall, precipitation of minerals and polysaccharides, or extracellular binding through intracellular sequestration of metallothionein are a few examples of how these mechanisms may contribute to fungal lithium tolerance [10]. In the case of lithium, researchers are often concerned with the accumulation of this element and its availability compared to lithium carbonate, which is used in psychiatric treatment. In comparison to the psychiatric medication containing Li_2_CO_3_, high mineral content in fungal biomass was linked to greater availability in sequential extraction and in vitro digestion [88]. More encouraging outcomes were obtained in the case of *Hericum erinaceus*. As shown by the calculations, consumption of 100 g of dry-weight fruiting bodies grown in an environment containing 1 mM Li would equal 69% of the currently advised daily allowance of Li [53].

### 6.5. Copper

A number of enzymes depend on copper for their essential roles in numerous metabolic processes. The most typical signs of a copper shortage include anaemia, lethargy, and a lowered white blood cell count [49]. There is very little information on the mechanisms of copper bioavailability in the biofortification process. It is critical to understand the degree of bioavailability. Solubility is a significant element influencing bioavailability, even though the two cannot be equated. The more soluble forms of Cu are thought to be more potentially bioavailable than the less soluble forms. Furthermore, a substance must be present in the intestinal fluid in a soluble form—either as a free ion or as a chelate with another nutrient—in order for the body to absorb and possibly use it. Therefore, the bioavailability of both dry mycelium and supplements was evaluated based on the solubility of Cu in the simulated gastrointestinal tract [38].

## 7. Agronomic Considerations

Mushrooms can accumulate various mineral elements, such as selenium, zinc, copper, magnesium, manganese, and molybdenum, making them an important source of micronutrients essential to humans [89]. The concentration of minerals in mushrooms is affected by the ecological role, phylogenetic affinity, and environmental factors, enabling the maximisation of mineral content in harvested mushrooms by collecting them in specific areas. Mushrooms are low in energy and fat but high in protein, carbohydrates, dietary fibre, and a variety of minerals and trace elements, such as potassium, copper, iron, and selenium, making them a nutritious food with potential health benefits [90]. The mineral composition of mushrooms is influenced by the content of macronutrients in the soil, with the uptake of potassium, magnesium, and calcium by mushrooms being positively correlated with the content of these elements in the soil [91].

Furthermore, depending on the fungus species, different types of culture media are used. The substrate is important for bioavailability, interactions with substrate components and mycelium, and effects on fungal mass and growth. A major challenge in culturing fungi for biofortification is the correct choice of the concentration and form of the element added to the medium. Therefore, each case must be treated individually. In order to achieve optimum efficiency, the species to be enriched must be selected, and the ideal conditions must be determined. For example, Budzyńska et al. studied the effects of three forms of iron: FeCl_3_ 6H_2_O, FeHBED, and FeSO_4_ 7H_2_O applied at three concentrations (5, 10, or 50 mM) on three fungal species: *Pleurotus ostreatus, P. eryngii,* and *Pholiota nameko*. The concentration of 50 mM proved to be the best concentration for the species tested. The most favourable addition was FeHBED for *P. ostreatus* and FeCl_3_ 6H_2_O for *P. nameko* and *P. eryngii*. Additionally, *P*. *nameko* showed the highest accumulation of Fe among the three species [6]. Mleczek et al. studied three fungal species, *Pleurotus eryngii, Ganoderma lucidum*, and *P. ostreatus,* on media enriched with lithium (0.25–1.0 mM) in the form of carbonate or acetate. Lithium accumulation, fruiting body production, and mycelial colony growth were assessed. Li_2_CO_3_ turned out to be the most bioavailable form but at a higher cost to fungal development. The growth retardation was lessened or absent when CH_3_COOLi was added to the substrate, while the Li absorption was decreased. The most encouraging outcomes were achieved with *G. lucidum*, which accumulated up to 73.58 ± 10.87 (Li_2_CO_3_) and 25.59 ± 9.98 (CH_3_COOLi) mg Li kg^−1^ dry mass. The concentrations of Li accumulated in fruiting bodies were not high enough for application in psychiatric treatments but could potentially support the daily intake of Li for behaviour modification or health beneficiary purposes [88]. The most difficult and time-consuming task is the optimisation of the biofortification process. Even if the concentration and form of an element are the same, fungi grow on different substrates, and it turns out that the bioavailability of elements varies. The possible industrial application of biofortified mushrooms as nutraceuticals requires the economical and efficient production of biomass during their growth. The development and appearance of the mycelium can be greatly influenced by an artificial increase in the content of certain components in the growing media. This may lead to higher cultivation expenses, a decline in the end product’s appeal to potential buyers, and a reduced market value. Budzyńska et al. found no differences in colour and size between control fruiting bodies and fungi enriched with different concentrations of individual Fe salts. A similar pattern was seen in their biomass. Fungal yields were neither substantially greater nor lower in response to the increased Fe concentrations [40]. Oyetayo et al. reported the same observation, as the addition of 500 mg kg^−1^ did not reduce the yield of *Pleurotus pulmonarius* [48]. Similarly, Meniqueti et al. and Almeida et al. found no significant inhibition of fungal growth at lower concentrations of up to 70 mg L^−1^ and up to 150 mg L^−1^, respectively [24,34]. In contrast, Umeo and co-authors examined 14 strains of *Agaricus subrufescens*, and 12 were characterised by their lower biomass production after the addition of 50 mg L^−1^ Fe [35]. Biofortification of mushrooms using fermentation processes such as solid-state fermentation (SSF) and submerged fermentation (SmF) allows the enrichment of mushrooms with various elements, but the effects vary depending on the method. In SSF, fungi grow on a solid medium, and it allows for a more natural way of growth, which often better promotes biofortification in minerals such as selenium, iron, or zinc. In SmF, on the other hand, the fungi grow in a liquid medium, which allows for faster growth and easier control of fermentation conditions. But, the elemental forms may be less bioavailable, and mineral concentrations are sometimes lower compared to SSF. However, SmF may be better for enrichment in some specific elements or compounds. Fermentation processes can significantly affect the efficiency of biofortification depending on the type of fungus, the element used, and the form of its introduction [92]. Submerged fermentation, for example, is used to enrich fungi such as Antrodia camphorata with selenium. In this process, the fungus effectively converts sodium selenate into organic forms [93]. Solid-state fermentation, on the other hand, is often used to biofortify soybeans using edible fungi such as Pleurotus ostreatus. SSF allows for significant improvements in the nutritional and antioxidant values of substrates. Differences between these processes can affect how fungi accumulate micronutrients and convert them into bioactive forms, which is key to designing functional foods [94].

## 8. Challenges and Future Directions

**Figure 3:**
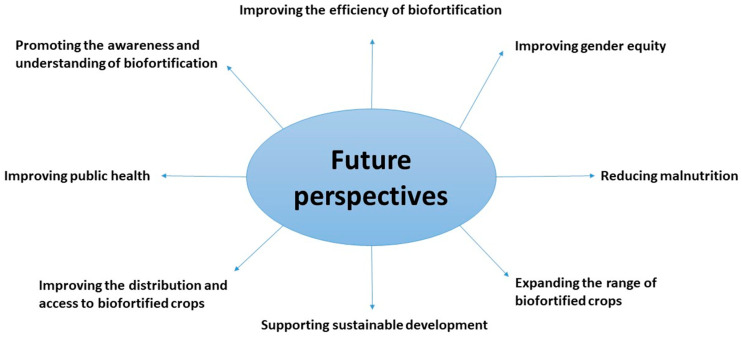
Future perspectives for biofortification.

The need for nutrient-rich food is rising along with the world population, making biofortification an increasingly significant field of study. Researchers are trying to create new crops with higher concentrations of vital vitamins and minerals. A growing number of nutrient-rich crop types that can aid in addressing global hunger and enhancing public health are probably going to be developed as research in this area progresses. Figure 3 illustrates some future perspectives on biofortification [20].

**Figure 4:**
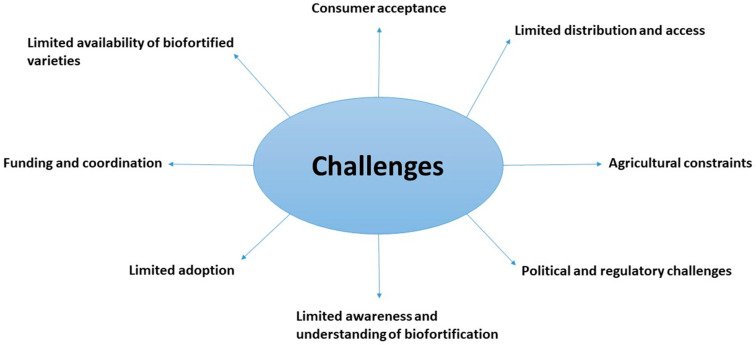
Challenges for biofortification.

The aim of effective biofortification should be to increase the concentration of micronutrients while also boosting their bioavailability. This can be accomplished by lowering the concentrations of nutrients that obstruct absorption and raising the concentrations of compounds that increase the absorption of minerals. Vitamins D, C, and E, as well as provitamin A, choline, and niacin, are substances that stimulate the absorption of methionine, tryptophan, Zn, Fe, P, Ca, and Se. In contrast, some polyphenols and phytates reduce the bioavailability of micronutrients. Phytate is a form of phosphorus stored in seeds. During digestion, it can bind with zinc and iron and thus reduce absorption. The phytate concentration can be controlled by manipulating phytate biosynthesis through mutation of the myo-inositol kinase (MIK) gene, identification of low-phytate lines through embryo screening, and overexpression of phytase, i.e., a phytate-degrading enzyme. Figure 4 illustrates some challenges for biofortification [3].

The analysis of the literature revealed that the research carried out in the field of biofortification is focused on the determination of the effects of various micronutrients and macronutrients, while it is difficult to find articles analysing other chemical compounds, such as vitamins. Another interesting compound that has a number of health-promoting properties is gamma-aminobutyric acid (GABA). This non-protein amino acid acts as a major inhibitory neurotransmitter in the central nervous system. A good innovative trend in biofortification that would be worthwhile to introduce is the study of biofortified products on cell lines. In this way, it would be possible to check whether a particular compound has a beneficial effect on normal cells or an inhibitory effect on the proliferation and viability of cancer cells. Changes at the molecular level and changes in the secretory functions of tissues are also assessed. The problem with in vitro studies is the direct translation of the results obtained into a complex in vivo system. However, despite the simplistic approach in cell cultures to assessing the activity of compounds, in vitro studies allow, e.g., the determination of the doses of a substance or the time of exposure to a compound necessary for the desired effects to appear in cells. The advantages of using cell cultures include the possibility to perform tests on different cell types, the low cost of the tests, the reproducibility and ease of analysis, the possibility to perform tests with a small number of substances, and the accurate and relatively fast analysis of changes in cells [95].

## 9. Conclusions and Perspectives

Around 2 billion people worldwide suffer from micronutrient deficiencies. “Hidden hunger” is a fairly common phenomenon in developing countries. It is a phenomenon in which the organism lacks the minerals and vitamins needed for development, growth, and good overall health. Deficits in these phytochemicals can have major health repercussions, including decreased cognitive function in children, greater risk of infection, and a host of other detrimental effects on physical and mental health [96]. The process of biofortification is a strategy developed to combat hidden hunger. It involves increasing the levels of essential nutrients in crops that are commonly consumed by humans. There are several methods of biofortification. While transgenic approaches provide long-term solutions, they take time to produce nutrient-rich varieties. In contrast, agronomic approaches provide short-term immediate solutions. As can be seen, crop enrichment has many advantages and can bring many benefits, but it has to overcome various challenges to become a widespread technique, as very few crops have been commercialised to date [97]. Mushrooms are increasingly being considered for biofortification due to their ability to accumulate various elements. Therefore, they can be a good option for enriching foods with micro- and macroelements, but this still requires further research [98]. Future research worth considering when analysing biofortification is the fungal metabolome, which can show how this process affects fungal metabolism. Another important consideration is the examination of an organoleptic sensory array by a consumer panel so that it will be possible to assess how biofortification affects the flavour and aroma characteristics and studies related to the consumption of biofortified mushrooms by the population/persons who do not have such nutritional needs. The possibility of targeted biofortification aimed at adding such chemical compounds to the mushroom to achieve the desired properties can also be explored.

## Figures and Tables

**Table 1:** Nutrients of selected edible mushrooms and commonly consumed foods. Own compilation based on FoodData Central, USDA. The content of individual nutrients is given per 100 g of fresh weight of the product.

Chemical Compounds	Agaricus Bisporus	Lentinula Edodes	Pleurotus Eryngii	Pea	Rice	Broccoli	Avocado	Chicken Breasts	Bread	Sweet Potato
Protein	3.09 g	2.24 g	2.41 g	5.42 g	2.69 g	2.82 g	2 g	23.2 g	10.7 g	1.57 g
Total dietary fibre	1 g	2.5 g	3 g	5.7 g	0.4 g	2.6 g	6.7 g	-	4 g	3 g
Vitamin C	2.1 mg	-	-	40 mg	-	89.2 mg	10 mg	1.1 mg	0.2 mg	2.4 mg
Iron, Fe	0.5 mg	0.41 mg	0.34 mg	1.47 mg	1.2 mg	0.73 mg	0.55 mg	0.64 mg	3.6 mg	0.61 mg
Sodium, Na	5 mg	9 mg	1 mg	5 mg	1 mg	33 mg	7 mg	67 mg	473 mg	55 mg
Calcium, Ca	3 mg	2 mg	2.49 mg	25 mg	10 mg	47 mg	12 mg	18 mg	125 mg	30 mg
Magnesium, Mg	9 mg	20 mg	13.5 mg	33 mg	12 mg	21 mg	29 mg	-	41 mg	25 mg
Potassium, K	318 mg	304 mg	294 mg	244 mg	35 mg	316 mg	485 mg	-	141 mg	337 mg
Zinc, Zn	0.52 mg	1.03 mg	0.63 mg	1.24 mg	0.49 mg	0.41 mg	0.64 mg	-	1.04 mg	0.3 mg
Copper, Cu	0.32 mg	0.142 mg	0.47 mg	0.176 mg	0.069 mg	0.049 mg	0.19 mg	-	0.148 mg	0.151 mg
Selenium, Se	9.3 µg	5.7 µg	1.2 µg	1.8 µg	7.5 µg	2.5 µg	0.4 µg	-	28.8 µg	0.6 µg

**Table 2:** Species of mushrooms used in the biofortification process.

Species of Mushroom	Biofortified Chemical Compound	Literature
*Cordyceps militaris*	Selenium (Se)	[23]
*Lentinus crinitus*	Iron (Fe)	[24,25]
*Coriolus versicolor*	Selenium (Se)	[26]
*Pleurotus ostreatus*	Iron (Fe), Zinc (Zn), Lithium (Li), Selenium (Se), Calcium (Ca), Germanium (Ge)	[27] [6] [10,25] [28] [29] [30] [31,32,33] [9,34,35,36,37]
*Grifola frondosa*	Copper (Cu), Zinc (Zn), Selenium (Se)	[38,39]
*Pholiota nameko*	Iron (Fe), Calcium (Ca)	[6,40]
*Pleurotus eryngii*	Iron (Fe), Selenium (Se), Zinc (Zn), Calcium (Ca)	[6,41] [25,29] [9,31,32,35]
*Ganoderma lucidum*	Iron (Fe), Selenium (Se), Calcium (Ca), Copper (Cu), Zinc (Zn), Germanium (Ge)	[25,29] [32,42] [33,43] [9]
*Schizophyllum commune*	Iron (Fe), Zinc (Zn)	[25,29] [35]
*Lentinula edodes*	Iron (Fe), Selenium (Se), Zinc (Zn)	[25] [44] [9,35]
*Agaricus subrufescens*	Iron (Fe)	[25,29]
*Pleurotus florida*	Selenium (Se), Zinc (Zn)	[9,30,45]
*Pleurotus sajor caju*	Selenium (Se), Zinc (Zn)	[9,30,46]
*Pleurotus djamor*	Selenium (Se), Zinc (Zn), Calcium (Ca)	[9,32,46] [47]
*Pleurotus pulmonarius*	Zinc (Zn), Iron (Fe), Copper (Cu), Lithium (Li), Selenium (Se)	[9,36,47] [48] [49]
*Pleurotus citrinopileatus*	Zinc (Zn)	[47]
*Flammulina velutipes*	Selenium (Se), Calcium (Ca)	[32,50]
*Hericium erinaceus*	Selenium (Se), Lithium (Li)	[42,51] [52] [53]
*Pleurotus floridanus*	Calcium (Ca)	[54]
*Hypsizygus marmoreus*	Calcium (Ca)	[32]
*Inonotus obliquus*	Calcium (Ca)	[32]
*Pleurotus nebrodensis*	Calcium (Ca)	[32]
*Poria cocos*	Calcium (Ca)	[32]
*Laetiporus sulphureus*	Calcium (Ca)	[32]
*Agaricus bisporus*	Selenium (Se), Zinc (Zn), Copper (Cu)	[9,55]
*Agrocybe aegerita*	Selenium (Se)	
*Agrocybe cylindracea*	Lithium (Li)	[53]
*Pleurotus cornucopiae*	Selenium (Se)	[9]
*Pleurotus fossulatus*	Selenium (Se)	[9]
*Pleurotus citrinopielatus*	Selenium (Se)	[9]
*Volvariella volvacea*	Selenium (Se)	[9]
*Calocybe indica*	Selenium (Se)	[9]

## Data Availability

The original contributions presented in this study are included in the article.
